# Cellular automata modeling depicts degradation of cellulosic material by a cellulase system with single-molecule resolution

**DOI:** 10.1186/s13068-016-0463-8

**Published:** 2016-03-08

**Authors:** Manuel Eibinger, Thomas Zahel, Thomas Ganner, Harald Plank, Bernd Nidetzky

**Affiliations:** Institute of Biotechnology and Biochemical Engineering, Graz University of Technology, NAWI Graz, Petersgasse 12, 8010 Graz, Austria; Institute for Electron Microscopy and Nanoanalysis, Graz University of Technology, Steyrergasse 17, 8010 Graz, Austria; Graz Centre for Electron Microscopy, Steyrergasse 17, 8010 Graz, Austria; Austrian Centre of Industrial Biotechnology, Petersgasse 14, 8010 Graz, Austria

**Keywords:** Cellulose, Cellulase, System-level modeling, Cellular automata, AFM imaging, Hydrolysis, Surface degradation

## Abstract

**Background:**

Enzymatic hydrolysis of cellulose involves the spatiotemporally correlated action of distinct polysaccharide chain cleaving activities confined to the surface of an insoluble substrate. Because cellulases differ in preference for attacking crystalline compared to amorphous cellulose, the spatial distribution of structural order across the cellulose surface imposes additional constraints on the dynamic interplay between the enzymes. Reconstruction of total system behavior from single-molecule activity parameters is a longstanding key goal in the field.

**Results:**

We have developed a stochastic, cellular automata-based modeling approach to describe degradation of cellulosic material by a cellulase system at single-molecule resolution. Substrate morphology was modeled to represent the amorphous and crystalline phases as well as the different spatial orientations of the polysaccharide chains. The enzyme system model consisted of an internally chain-cleaving endoglucanase (EG) as well as two processively acting, reducing and non-reducing chain end-cleaving cellobiohydrolases (CBHs). Substrate preference (amorphous: EG, CBH II; crystalline: CBH I) and characteristic frequencies for chain cleavage, processive movement, and dissociation were assigned from biochemical data. Once adsorbed, enzymes were allowed to reach surface-exposed substrate sites through “random-walk” lateral diffusion or processive motion. Simulations revealed that slow dissociation of processive enzymes at obstacles obstructing further movement resulted in local jamming of the cellulases, with consequent delay in the degradation of the surface area affected. Exploiting validation against evidence from atomic force microscopy imaging as a unique opportunity opened up by the modeling approach, we show that spatiotemporal characteristics of cellulose surface degradation by the system of synergizing cellulases were reproduced quantitatively at the nanometer resolution of the experimental data. This in turn gave useful prediction of the soluble sugar release rate.

**Conclusions:**

Salient dynamic features of cellulose surface degradation by different cellulases acting in synergy were reproduced in simulations in good agreement with evidence from high-resolution visualization experiments. Due to the single-molecule resolution of the modeling approach, the utility of the presented model lies not only in predicting system behavior but also in elucidating inherently complex (e.g., stochastic) phenomena involved in enzymatic cellulose degradation. Thus, it creates synergy with experiment to advance the mechanistic understanding for improved application.

**Electronic supplementary material:**

The online version of this article (doi:10.1186/s13068-016-0463-8) contains supplementary material, which is available to authorized users.

## Background

Cellulose is a water-insoluble linear polysaccharide composed of several hundred or more β-1,4-linked D-glucosyl units. Its depolymerization by hydrolytic enzymes occurs through repeated endo- and exo-type cleavages inside of and from the ends of the cellulose chain, respectively [1, 2]. Regarding only a single chain, therefore, the enzymatic hydrolysis might appear as a fairly simple transformation. However, from a system-level viewpoint, degradation of cellulosic material represents an extremely complicated process [1–7]. Its kinetics involves an array of heavily entangled, enzyme- and substrate-related complexities appearing at different length scales in dependence of time and conversion [5, 8–14]. Despite much progress made in delineating function of individual elements [1–3, 15–22], coherent description of the degradation process as a whole proved elusive. Bottom-up reconstruction of total cellulose/cellulase system behavior therefore constitutes an increasingly important research aim of high fundamental but also practical significance.

**Scheme 1:**
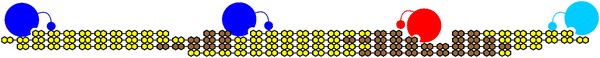
A typical set of fungal cellulases acting on mixed amorphous–crystalline cellulose. Chain end-cleaving cellobiohydrolases (CBH I *blue*; CBH II *cyan*) and internally chain-cleaving endoglucanases (*red*) catalyze the hydrolysis of crystalline (*yellow*) and amorphous (*brown*) cellulosic material

Core problem of enzymatic cellulose degradation in the way analyzed herein is to depict the spatiotemporally correlated action of distinct polysaccharide chain-cleaving cellulase activities confined to the dynamically evolving surface of the insoluble substrate. Progress in this effort is restrained severely by difficulties in unraveling interfacial enzyme catalysis experimentally. The problem in general is exacerbated in the case of the cellulose–cellulase system due to the fact that the actual substrate is a solid material of typically high morphological irregularity [1, 3, 9]. Depending on cellulose source and isolation procedure, there is variation and uneven distribution in the spatial arrangement of the polysaccharide chains [1, 5, 9]. Parts of the cellulose that feature disorder in position, direction, or orientation of the chains therefore alternate with highly organized, crystalline material. Most of the cellulose chains are initially not accessible to the enzymes due to the surrounding material [5]. Individual cellulases differ in preference for degrading crystalline compared to amorphous cellulose [1, 2, 7, 14]. Crystalline cellulose is degraded primarily through chain ablation promoted by exo-cellulases acting in a processive manner (Scheme 1) [16, 19]. Degradation of amorphous material additionally involves internal disruptions of cellulose chains by endo-cellulases (Scheme 1) [13, 14]. The spatial distribution of structural order across the cellulose surface therefore imposes constraints on the attack by different cellulases and also on the dynamic interplay between the enzymes. What results therefore is a highly uneven distribution in the local rates of removal of surface material by lateral and vertical degradation [14]. Morphology and also shape of the cellulose surface thus undergo large spatiotemporal changes as the conversion makes progress [14, 15, 23], with consequent reciprocal action on the chain fragmentation activity by the adsorbed enzymes. While providing a convenient measure of the overall cellulose degradation efficiency, release of soluble sugars is a frequently used but highly convoluted parameter that does not support direct inference to cellulase action on the evolving cellulose surface. Owing to limitations in resolving interfacial processes by experiment, mathematical modeling has traditionally played a leading role in developing the mechanistic thinking of cellulose degradation [5, 8–12, 24–35].

Paradigmatic approach of modeling enzymatic cellulose degradation involves development of a set of differential equations describing the relevant physical and chemical steps of the reaction (e.g., cellulase adsorption, chain fragmentation) under the constraints of mass balance [5, 11]. Despite notable recent progress [11, 12, 25, 26], mathematical representation of the substrate’s morphology and the change thereof with time and conversion in mutual dependency on the chain fragmentation activity of the locally adsorbed cellulases is at the very limit of what current mass balance-based models are capable of delivering. Spatial heterogeneities in cellulose morphology can only be implemented in the form of distribution functions [12, 25, 26], but not explicitly for individual substrate samples. Model validation including determination of unknown parameters is therefore limited to comparison with lumped experimental data that lack spatial resolution.

Cellular automata (CA) models present an interesting alternative by virtue of their ability to literally depict the spatiotemporal behavior of complex systems across length scales [36]. CA models constitute mathematical systems constructed from many small entities, each simple and obeying preassigned rules, but together capable of complex performance. Evidence from CA modeling is suitable for visual inspection, and what is most important, it offers the unique opportunity to be validated against time and laterally resolved data from advanced imaging analysis. Visualization by real-time AFM [13, 14, 16, 19] and high-resolution fluorescence spectroscopy [6, 7, 17, 18] has recently provided unprecedented insight into the dynamics of cellulose degradation at different length scales down to single-enzyme resolution. Therefore, this emphasizes the need but also the potential of a modeling approach optimally aligned to the capabilities of the emerging visualization techniques. CA modeling has already been applied to the study of enzymatic hydrolysis of cellulose in a few earlier papers [30, 31, 36–39], but the important link to experimental visualization as a validation tool has not been established.

Using CA-based formalism, comprehensive system-level modeling of cellulose degradation by cellulases is reported. The herein developed substrate model allowed for realistic representation of amorphous–crystalline cellulosic material previously applied by this group of authors in AFM imaging experiments [14]. The enzyme model incorporated the three main activities of non-complexed fungal cellulase systems: two processively chain-end cleaving cellobiohydrolases (CBHs) acting from the reducing (CBH I) and non-reducing (CBH II) end of the cellulose chain, respectively; and an internally chain cleaving endoglucanase (EG). CBH I was known from experiment to attack crystalline material, whereas CBH II and EG degraded amorphous substrate parts [14]. We show in simulations, here for the first time, that observable characteristics of cellulose surface degradation by the synergizing set of cellulases (e.g., local surface height degradation rates; surface morphology evolution) were reproduced in useful quantitative agreement with the experiment [14]. Interestingly, the simulations also reproduced a counterintuitive experimental finding: once uncovered from obstructing amorphous material, crystalline nanofibrils were degraded significantly faster than supposedly less resistant amorphous cellulose in their surrounding [14]. Simulations also revealed that slow release of cellulose chains by CBH II resulted in pronounced jamming of processively acting enzymes at the interface of the amorphous and crystalline phases. In addition to the intrinsically slow kinetics of CBH I action [20, 35, 40], this was clear additional factor of rate limitation in the degradation of crystalline cellulose as simulated. The model might aid in the elucidation of inherently complex phenomena of cellulose degradation and thus create synergy with experiment to advance the mechanistic understanding as well as the application.

## Results and discussion

### A CA model of mixed amorphous–crystalline cellulose

**Fig. 1:**
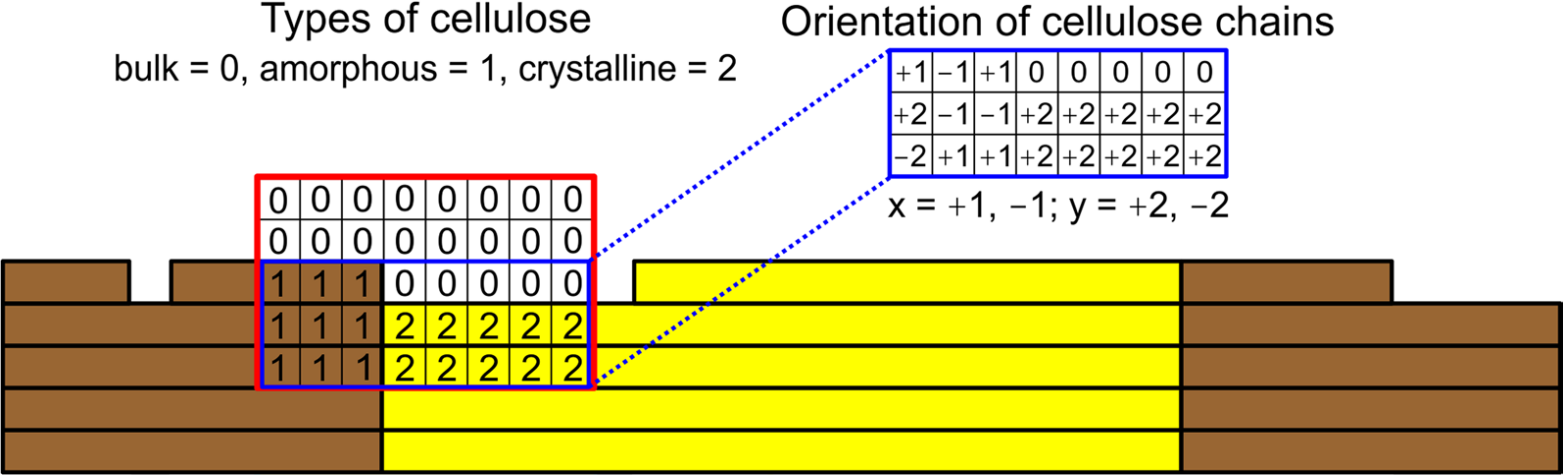
CA model of mixed amorphous–crystalline cellulose. Cellulose material is modeled as a large 3D matrix (depicted here in a schematic 2D *y*–*z* view) composed of *substrate automata* that represent cellobiose units. Tokens ‘1’ and ‘2’ are used to indicate *cellobiose units* in amorphous and crystalline material environment. Token ‘0’ is used to indicate *bulk molecules*. The corresponding matrix is framed *red*. Further properties of the cellulose chains such as the orientation in space and the location of the hydrophobic faces are assigned through additional matrices of identical size, as shown in the matrices framed in *blue*. Each cellobiose unit in the cellulose model is identified unambiguously through the positions in all substrate matrices

**Fig. 2:**
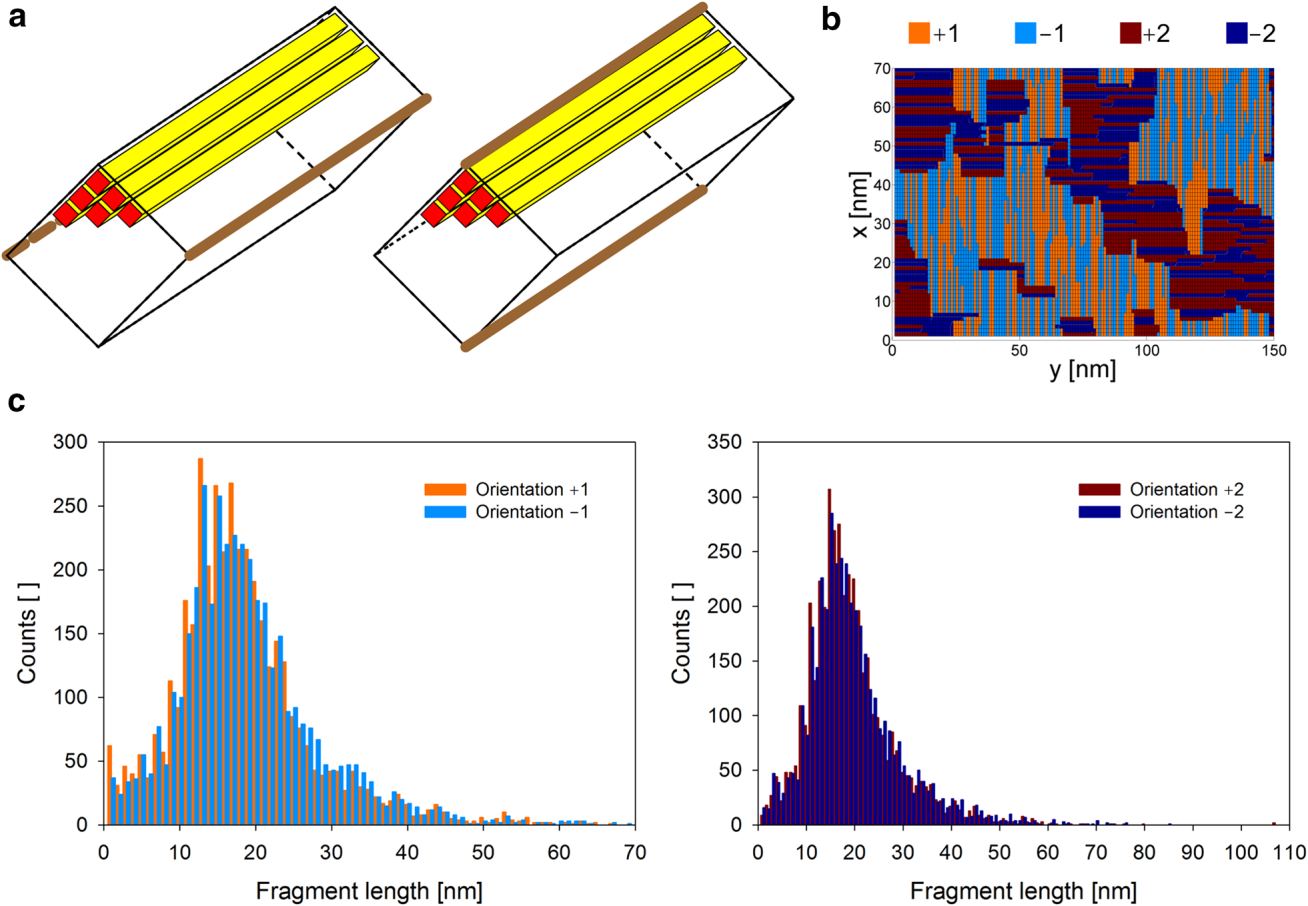
Structure of crystalline (**a**) and amorphous (**b**) cellulose as implemented in the CA model. **a** Cellulose nanocrystals were modeled in analogy to the natural elementary fibril of cellulose allomorph I and were composed of up to 144 cellulose chains (16 nm crystals). Their length was constant at 100 nm equaling 100 cellobiose units per chain, and the width/height varied between 8 and 16 nm. *Yellow bars* show the cellulose chains with their reducing end indicated in *orange*. Shown in *brown* are the so-called hydrophobic faces of the cellulose nanocrystal which is where attack by CBH I takes place. Two orientations of the nanocrystal within amorphous material are considered whereby the crystal’s hydrophobic faces are aligned horizontally (*left*) or vertically (*right*). **b** A random positioning algorithm (Additional file 1) was used to create amorphous cellulose layers with a final mean fragment length of 18 cellobiose units. The figure shows an exemplary amorphous plane. It also shows how amorphous chains with varying orientation are combined in one plane. Amorphous chains can be oriented in *x* or *y* direction in one *z*-plane. Tokens ‘±1’ and ‘±2’ are used within the amorphous chain orientation matrix to distinguish *x* and *y* direction, respectively. The internal direction of an amorphous chain is indicated by an algebraic sign (‘+’ or ‘−’) and relative to the origin of the *z*-plane (0, 0). A chain oriented from the reducing to the non-reducing end with respect to the origin has a positive sign (‘+’) and a vice versa oriented chain is indicated by a negative sign (‘−’). Amorphous cellulose material was obtained by stacking multiple layers of amorphous cellulose on top of each other. **c** The distribution pattern for the cellulose chain lengths in 30 independent planes making up the amorphous material is shown for *x*- (*left panel*) and *y*-oriented (*right panel*) cellulose chains demonstrating the essentially random distribution of the chains in all orientations

Figure 1 shows a two-dimensional representation of the three-dimensional matrix model of the cellulosic substrate. Cellulose chains were coarse-grained with the dimer cellobiose as their smallest unit (assumed length: 1 nm). Similar approaches of modeling of cellulose chains have been described before [30, 31, 36, 37]. In the matrix system used, the crystalline and amorphous phases were identified through tokens 1 and 2, respectively, assigned to each cellobiose molecule comprised in the cellulose. Token 0 was used to indicate the liquid bulk phase. A secondary matrix assigned the cellulose chain orientation in space, as illustrated in Fig. 1. Other structural characteristics of the cellulose were assigned using additional matrices, as follows. Crystalline nanofibrils were modeled as cuboids (length: 100 nm; width/height: 8–16 nm) assembled from orientationally and directionally ordered cellulose chains (Fig. 2a). Their size corresponded approximately to nanofibrils of the real cellulose substrate used in experiments [41]. The internal chain organization was that of cellulose allomorph I that was known to account chiefly for the crystalline phases in the experimental substrate [41]. Figure 2a additionally shows the so-called hydrophobic faces of the nanofibril, indicating the crystalline surfaces where enzymatic attack takes place [19, 42]. A similar modeling concept was used recently to describe the orientation and accessibility of the hydrophobic faces in cellulose nanofibrils [30, 43]. Note: the possibility that individual cellulose chains of the hydrophobic face differ in “decrystallization energy” and therefore in accessibility to enzymatic attack, as has been suggested in in-depth modeling studies of the crystalline cellulose structure [32, 44], was not considered in our model. This is not an intrinsic limitation of the coarse-grained modeling approach used here. Individual crystalline cellulose chains on the hydrophobic face could be assigned distinct enzyme accessibility properties. However, in view of the focus of current study description of the crystalline phase down to the level of the individual chains’ “energy” was considered to be not necessary.

Amorphous cellulose has no unique structure by definition [45], and little is known from experiment about its structural organization. Any model of amorphous cellulose is therefore hypothetical. The idea pursued in this study was to represent the amorphous substrate as a material consisting of completely randomly organized cellulose chains. We have no way of knowing, however, how well this model represents the real amorphous cellulose used in the experiment. Algorithm was developed to create randomized orientation of fragments of cellulose chains in space (Fig. 2b). Fragments with varying length, mimicking a Gaussian distribution, were generated and placed in a 2D plane with random orientation in *x* or *y* direction. The internal direction of cellulose chains, from the reducing to the non-reducing end or from the non-reducing to the reducing end, was also considered and implemented in the model (Fig. 2b). A mean fragment length of 18 cellobiose units was obtained, corresponding well to the mean free path length reported from amorphous cellulose [46]. An example of how an amorphous cellulose plane was generated is shown in Additional file 1. The modeled cellulose chain length was 150 cellobioses, representing the estimated degree of polymerization of the experimental substrate derived from microcrystalline cellulose (Avicel) [37, 47, 48]. Figure 2c shows that chain fragment orientations were normally distributed across different planes analyzed, confirming a completely random structural organization of the modeled amorphous material. Each field of amorphous cellulose therefore has a probability of 2/150 to be a free chain end. The free chain ends comprise equal portions of reducing and non-reducing chain-end types. We are well aware of the fact that our cellulose model presents a strong simplification. Note, therefore, that a more detailed distinction and further diversification of cellulosic material would be possible in the model. One way of achieving this would be through a more sophisticated representation of the substrate structure and by introducing multiple complexation rate constants [11, 30, 49], which are dependent on the organization of the cellulosic substrate and the type of attacking enzyme. However, comparing the physical dimensions of our CA model (see the “Methods” section) and the estimated degree of polymerization of cellulose (as stated above), it is not likely that we have multiple cellulosic phases present at the same time. Thus, we decided to reduce the complexity of the substrate to a two-phase substrate model (crystalline and amorphous).

### Basic CA model of the cellulases and their action on the cellulose surface

**Fig. 3:**
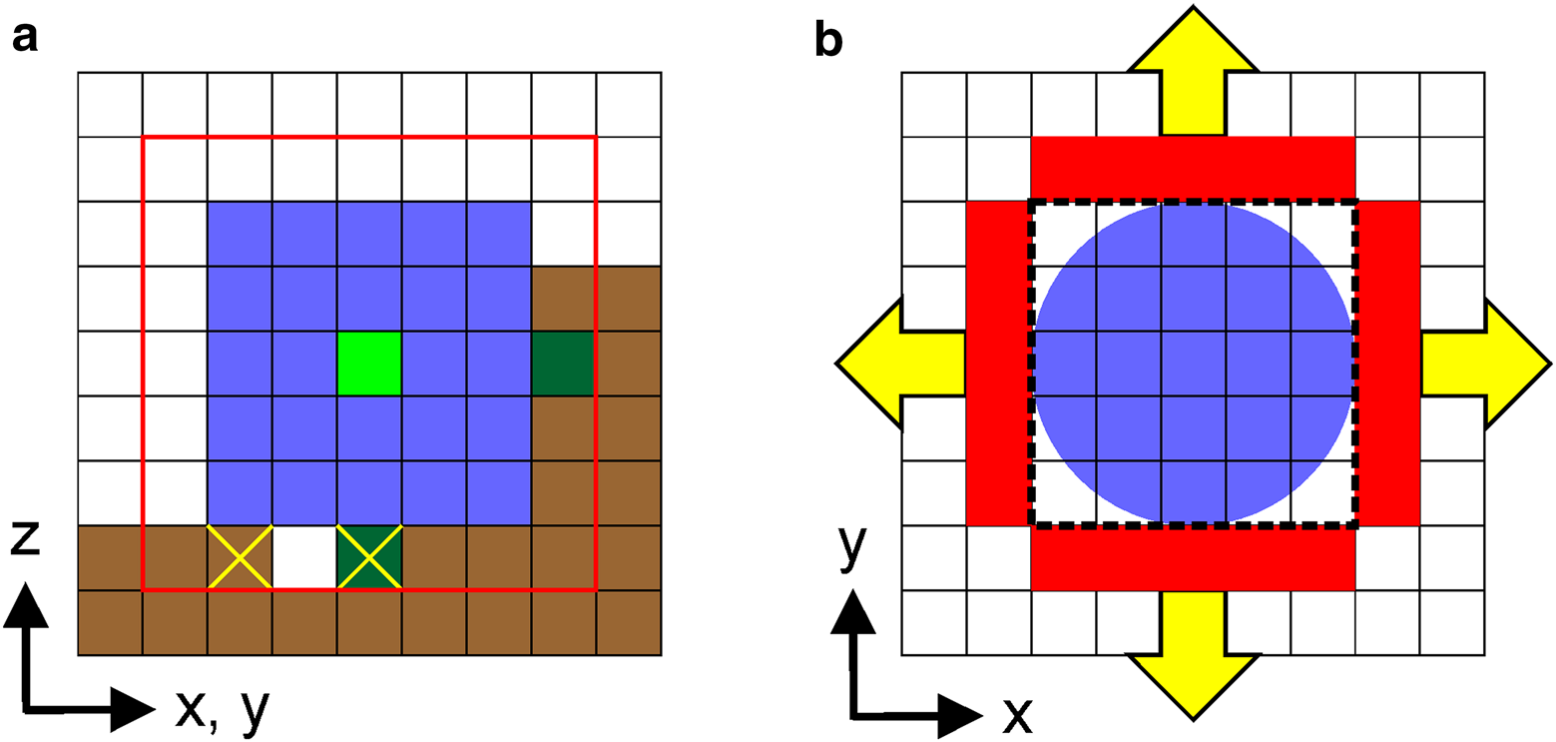
The CA model of the cellulases interacting with and degrading the cellulose surface. **a** The footprint of the enzyme is shown in *blue* and the center of the enzyme is shown in *light green*. The center of an enzyme is set within a distance of three water or cellobiose units to a randomly chosen point on the cellulosic surface (one of two *dark green squares*). Enzymes screen for substrate sites in their immediate surrounding (*red box*). For EG, any cellobiose (*brown*) of amorphous cellulose is a substrate, whereas for the cellobiohydrolases, only cellobioses lacking a neighbor cellobiose (indicated with a *yellow cross*) are substrates. **b** Top view of a CBH molecule in processive motion is shown. The wall in front of the enzyme in moving direction (*red rectangle*) is checked for structural obstacles (cellobiose molecules). The enzyme is represented against other enzymes as a sphere (*blue circle*) to calculate collision events. If neither substrate nor enzyme obstacles are present, the cellobiohydrolase moves along the chain, cleaving one cellobiose per 1/*k*_cat_ time interval

All cellulases were modeled as cubes (while interacting with cellulose) and as spheres (while interacting with other enzymes), as shown in Fig. 3. The edge length or diameter was 5 nm so that the modeled enzyme size was approximately that of the catalytic modules of cellulases from the fungus *Hypocrea jecorina* (formerly *Trichoderma reesei*) [2, 50]. In reality, each catalytic module is flexibly linked to a cellulose-binding module of considerably smaller size (~2 nm), as depicted in Scheme 1. By consolidating the two-module structure–function principle of cellulases into a single cellular automaton, the enzyme model was computationally efficient. We emphasize that the aim of the work was to describe the behavior of several hundreds of cellulases acting together, not that of a single enzyme. Model refinement to account for a two-module cellulase was therefore considered unnecessary. Based on the assumed footprint of a bound cellulase, requirements for accommodating the enzymes on the cellulose surface are depicted in Fig. 3a. The light green enzyme’s center is set within a distance of three water or cellobiose units to a randomly chosen point on the cellulosic surface (indicated in dark green). For enzyme placement to be allowed the adsorption footprint on the surface must be completely flat, that is, it must not contain any protruding cellobiose units. Furthermore, distance rules apply concerning the presence of other enzymes. A sphere with a diameter of 5.75 nm around the enzyme’s center point is calculated and must be available to the enzyme to be placed successfully. Thus, the attack of an enzyme on a cellobiose unit covered by another enzyme is avoided. For the initial adsorption, enzymes are positioned on the surface one after the other, starting with the set of EG molecules followed by the CBH I set and then the CBH II set. Note that enzyme placement of the cellulose is random not only regarding surface position but also surface material (amorphous, crystalline) and chain orientation.

In the current model focusing on *surface*-*bound* characteristics of cellulase action, enzyme adsorption was simplified to a quasi-irreversible process. It comprised a completely random positioning on the cellulose surface of a defined number of enzymes of each cellulase type, as will be described under *Programming, Simulation, and Data Processing* in more detail. Once positioned, enzymes ultimately remained surface bound over the entire time course of the simulation. This was effectuated by an immediate and again random re-positioning on the surface of any cellulase “released” from the surface.

This was modeled as a new round of random positioning on the cellulose surface of all enzymes except the ones being in “complexed” state, that is, the CBH acting processively on polysaccharide chains [22, 51]. Surface diffusion of the cellulases could be assumed from experimental evidence [18, 52, 53] to be at least 100 times faster than the overall process of chain cleavage (*k*_cat_). For each time span equal to 0.07 s (0.25/*k*_cat_ CBH I), therefore, the non-complexed enzymes were made to undergo randomization of their distribution on the surface. We considered that cellulase adsorption might directly target distinct substrate morphologies [54–56]. However, due to the relatively small surface areas (~1 × 10^4^ nm^2^) analyzed in our simulations, diffusional randomization superseded completely possible effects of enzyme specificity during cellulase adsorption to the cellulose surface. Computational rules for enzyme release were different for EG and the processively acting CBHs.

Based on their individual substrate requirements, the bound cellulases began searching for accessible cellulose chain types in immediate adjacency to them, as shown in Fig. 3a. For EG, any cellobiose of amorphous cellulose is a substrate, whereas for the CBH only cellobioses lacking a neighbor cellobiose are substrates (Fig. 3a). Cellobiohydrolase furthermore evaluates the free cellobioses for material property (crystalline, amorphous) and chain end type (non-reducing, reducing) to distinguish substrate sites for CBH I and CBH II. The enzymes thus became active in chain cleavage (modeled as replacement of a cellobiose by a water unit). The EG is then “released,” enabling it to become randomly re-positioned on the cellulose surface. The CBH screens the possibility to enter into the processive mode of action and, if possible, they remain “complexed” (attached to a cellulose chain) which prevents them from being redistributed on the surface. Once complexed, the CBH performed multiple cleavages on a single cellulose chain. Cellobiose was substituted by water on each cleavage, which occurred with time constant of 1/*k*_cat_. Processive action continued until an obstacle or chain end was encountered or the characteristic time of decomplexation (1/*k*_off_) had elapsed. Note that each CBH has an internal “stopwatch” to monitor if 1/*k*_off_ has already expired. The “stopwatch” is started once processive action is initialized and only reset if the enzyme is released from the surface. Structural obstacles are perceived in moving direction in a wall located in front of the enzyme (Fig. 3b). Cellobiohydrolases obstructed in their processive action due to obstacles encountered are required to wait a time corresponding to 1/*k*_off_ to switch back to the non-complexed (redistribution-able) state. Alternatively, blocked enzymes are allowed to continue processive action if the obstacle is removed. To account for a certain flexibility of both the enzymes [57, 58] and the threaded cellulose chain [59], the minimum size of a substrate obstacle obstructing processive motion of the CBH is defined as three cellobiose units.

Table 1 summarizes the *k*_cat_ and *k*_off_ values used in the final CA model of the cellulases. For CBH I, the *k*_cat_ [16] and the *k*_off_ [46] were taken from literature. The *k*_cat_ for CBH II and EG as well as the *k*_off_ for CBH II were not known and therefore used as adjustable parameters in a comparison of simulation results with experimental data (see “Results and discussion” section).

**Table 1:** Enzyme kinetic parameters used in the CA model of cellulase action

CA model parameter				
Enzyme	Type of cellulose material attacked	Type of chain cleavage	*k*_cat_^a^ [s^−1^]	*k*_off_^b^ [s^−1^]
CBH I	Crystalline	Exo	3.6^c^	0.7 × 10^−3d^
CBH II	Amorphous	Exo Endo	0.7^e^ 0.03	0.7 × 10^−2e^ –
EG	Amorphous	Endo	1.2^e^	–

^a^The *k*_cat_ is the rate constant for polysaccharide cleavage in exo-mode, endo-mode, or both

^b^The *k*_off_ is the rate constant of chain dissociation for a cellobiohydrolase acting in processive chain cleavage mode; its reciprocal (1/*k*_off_) is the time the cellobiohydrolase remains bound to the cellulose chain in such a mode of action event; an enzyme having *k*_off_ = 0 is perfectly processive, that is, it does not release the chain until it has reached its end. Therefore, in the case an obstacle is encountered during processive chain cleavage, this enzyme remains trapped until the obstacle is removed. Note that a cellobiohydrolase having *k*_off_ >0 is not an endoglucanase. It is an exo-acting enzyme that shows reduced processivity and is less likely to become trapped by obstacles to processive action

^c^Reference [16]

^d^Reference [46]

^e^Estimated on comparing results of CA modeling to experimental data of AFM imaging

Each chain cleavage by EG had to be modeled so that it produced both a reducing and a non-reducing chain end. Under framework conditions of the coarse-grained CA model of the cellulose chain (Fig. 1), it was convenient and also computationally efficient to have EG release one cellobiose for each cleavage performed, thereby creating the two types of chain end as required. In reality, according to the widely held view of EG action [1, 11, 60], this may not occur as the attack of EG on longer cellulose chains is thought to take place primarily by chain fission. However, we note that EG releases cellobiose in the experiment already at very early stages of the reaction which is difficult to explain by chain fission only. Moreover, EG might even exhibit a small degree of processivity as shown by Väljamäe and coworkers [46]. We therefore decided not to consider modeling of the EG as a pure chain-fission enzyme for mainly two reasons. First of all, it is not clear how such an enzyme can produce soluble sugars unless additional assumptions about the release of oligosaccharides from the solid material into solution are made. Furthermore, the additional algorithms needed to evaluate the length and the solubility of every surface-exposed cellulose chain in the system in each computational step are highly time-consuming. Secondly, the EG will not produce vertical degradation of the cellulose surface in the absence of further model assumptions and computational steps. In addition, we considered that random chain fragmentation by the EG ultimately results in the formation of cellobiose anyway. A possible error regarding the formation of soluble cellobiose was therefore small and the model of EG action was still considered suitable. The time course of cellobiose release was determined by calculating after each 7.0 s (i.e., a time span corresponding to 25 *k*_cat_ events of CBH I) the residual amount of all cellobiose units in the CA model and subtracting it from the initial amount of cellobiose units. We show that the time resolution of the simulated data was adequate and a more frequent readout of the residual amount of cellobiose units was therefore considered unnecessary.

### Degradation of amorphous cellulose by EG and CBH II

On incubation of the cellulose substrate with native *H. jecorina* cellulase or cellulase reconstituted from purified enzymes, amorphous material was removed from the surface with an average vertical degradation rate (*V*_z_) of 0.7 (±0.1) nm/min, as shown by AFM measurements [14]. According to our CA model supported from experiment [13, 14], attack on the amorphous cellulose is performed solely by EG and CBH II, whereas CBH I is not active on this material. We are sensible to the fact that strict categorization of the cellulases according to activity on amorphous or crystalline cellulose not only presents a strong simplification of what may be their true interaction with the insoluble substrate but is also at variance with some earlier observations made with these enzymes. CBH I was reported to be active on amorphous cellulose [21, 46] and CBH II showed degradation of crystalline cellulose [2, 61]. It should be noted therefore that first of all we refer to our own findings made with a special cellulosic substrate preparation [13, 14, 41]. Other celluloses used in earlier studies of cellulase specificity were different. Secondly, however, speaking of a purely amorphous or crystalline cellulose is difficult which leads to the problem of interpreting the measurements of soluble sugar release in terms of amorphous or crystalline material in the substrate having become degraded by the cellulase examined. The substrate used here was well characterized [13, 14, 41] and its degradation by the individual cellulases was measured not only by saccharification but also directly on the solid surface [13, 14]. Thirdly, for the purpose of modeling a clear-cut distinction between the different enzyme activities was considered useful. Finally, we believe that in the less strict sense, considering the effort in the modeling to represent the *main characteristic* properties of the individual cellulases, the CA models of CBH I, CBH II, and EG used here will not be controversial.

We started our modeling approach, therefore, by examining the actions of EG and CBH II, alone or in combination, on amorphous cellulose. Task of the modeling in particular was to determine the unknown *k*_cat_ values of the two enzymes. The cellulose substrate was modeled with a nanocrystal (16 × 100 × 16 nm) completely buried in amorphous material.

**Fig. 4:**
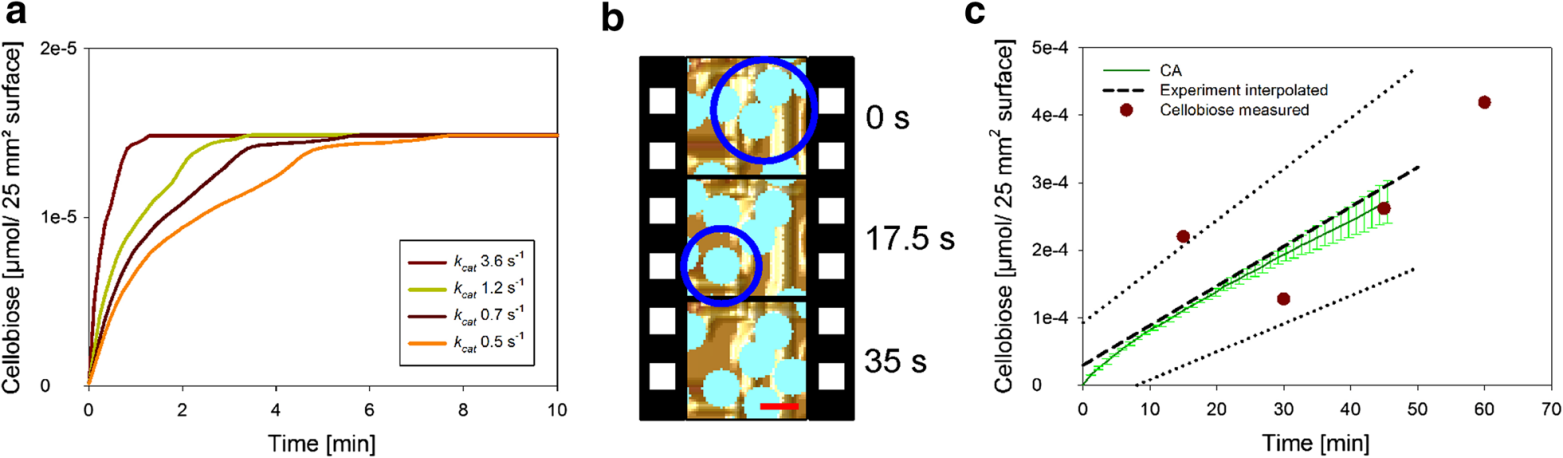
CA model of the action of CBH II on a mixed amorphous–crystalline cellulose substrate. The simulated substrate was a flat amorphous matrix in which a nanocrystal of 16 nm width/height and 100 nm length was embedded in plane. CBH II was placed in saturating amount on the cellulose surface (24 nmol/m^2^). **a** Effect of the enzyme’s *k*_cat_ on the time course of cellobiose released from 25 mm^2^ of cellulose surface. The same amorphous material was used on all simulations. Note that the rate of cellobiose release depends on the *k*_cat_, whereas the maximum amount of cellobiose does not. **b** When degradation of amorphous cellulose by CBH II (*light blue*) was simulated whereby CBH II was modeled as a perfectly processive exo-cellulase (*k*_off_ = 0), it was noted that CBH II became gradually trapped at amorphous material (*blue circle*). Collision between a complexed CBH II molecule and a structural obstacle (nanocrystal or amorphous material) was the origin of the jam, and a thus stuck CBH II presented an obstacle for other CBH molecules acting processively on amorphous cellulose chains nearby (*blue circle*). The *red scale bar* shows 5 nm. **c** Modeled time courses of cellobiose release by CBH II were not consistent with experiment unless the CA model of the enzyme was expanded to include endo-type chain cleavage in amorphous cellulose. *Modeled results* are shown for a *k*_endo_ of 0.03 s^−1^ and are compared with experimental data. The *k*_off_ of CBH II was set to 0.7 × 10^−2^·s^−1^ in the simulation (Table 1)

Simulations of individual enzyme reactions revealed that the *V*_z_ of EG was solely limited by the enzyme’s *k*_cat_. The *V*_z_ was independent of the number of chain ends accessible on the surface. We also noted and describe later that *V*_z_ of EG was unaffected by obstacles on the surface such as stuck CBH II molecules. CBH II alone, by contrast, showed low efficiency of surface material degradation. Its modeled *V*_z_ was hardly significant. This was explicable from the modeled action of the enzyme as a perfect exo-cellulase and, consequently, the absolute dependence of its activity on the availability of accessible non-reducing chain ends in the substrate. Limitation by accessible substrate sites is recognized in Fig. 4a where the modeled reactions of CBH II are shown to cease quickly with time. Progress curves of cellobiose release leveled out at a maximum of about 1.5 × 10^−5^ µmol cellobiose per 25 mm^2^ surface. Variation in the simulated *k*_cat_ of CBH II impacted on the reaction rate but not on the conversion of the substrate, as expected (Fig. 4a). We noted that the simulation results did not agree with experimental evidence, showing that CBH II was able to generate a significant *V*_z_ of up to 0.13 nm/min and to degrade up to 60 % of the cellulosic substrate used [13, 14]. Therefore, this was strong indication that the real action of CBH II was not that of a perfect exo-cellulase but also involved “EG-like” behavior to a certain degree. Literature also shows that CBH II might possess endo-cellulase activity [43, 46, 62, 63]. The CA model of CBH II was therefore expanded, assigning to the uncomplexed enzyme a certain probability of endo-type chain cleavage, expressed in the first-order rate constant *k*_endo_, while performing the diffusion-like “motion” on the cellulose surface. However, estimation of *k*_endo_ required knowledge of the *k*_cat_.

### Estimation of the *k*_cat_ of EG and CBH II

AFM experiments showed that EG activity alone was sufficient to cause substantial height degradation in amorphous material (*V*_z_ ≥0.4 nm/min). However, action of the EG also produced a “surface swelling” effect, which precluded determination of a well-defined *V*_z_ and thus assessment of the enzyme’s *k*_cat_ directly from surface imaging data of [13, 14]. The experimental *V*_z_ of 0.7 nm/min for the combined action of CBH II and EG restricted the *k*_cat_ of EG to an upper limit of 1.5 s^−1^, where the individual activity of the EG was already sufficient to produce the experimental *V*_z_. Note that the release of soluble sugars by EG under the relevant reaction conditions was not considered at this point, for it might strongly underestimate the actual turnover of the enzyme on the solid surface. EG, CBH II, and CBH I were therefore placed in a 3:1:1 ratio (see “Programming, simulation, and data processing” section) on the substrate to mimic the experimental conditions used [14] and CBH I was set inactive. Simulations were performed starting from a *k*_cat_ of 3.6 s^−1^ for CBH II, the same as that of CBH I [16], and a *k*_cat_ of 1.2 s^−1^ for EG. The endo-activity of CBH II was turned off (*k*_endo_ = 0) because, as long as EG was present in the system, the *k*_endo_ of CBH II was not a relevant factor of *V*_z_. The *k*_cat_ of EG was decreased in various steps until the simulated *V*_z_ dropped significantly below the experimental value. This sensitivity analysis suggested a *k*_cat_ of about ~1 s^−1^ for the EG. Tracking of the individual CBH II automata revealed that in the case of a perfectly processive CBH action (*k*_off_ = 0), the CBH II molecules became gradually trapped at places where other CBH II molecules or crystalline material presented obstacles for their further movement (Fig. 4b). We will show later that maintenance of surface dynamics in the action of CBH II via imperfect enzyme processivity (*k*_off_ >0) was essential for the CA model to reproduce characteristic spatiotemporal features of the substrate degradation by the complete cellulase system. Note that a CBH having *k*_off_ >0 is not an EG. It is an exo-acting enzyme that shows reduced processivity. This in turn enabled experimental validation of the *k*_off_. However, to obtain *k*_cat_ estimates for EG and CBH II, the plausible assumption was made first that the *k*_off_ for CBH II lies anywhere within two magnitude orders above or below the *k*_off_ for CBH I. Note that the final estimate of *k*_off_ clearly falls into this range.

**Fig. 5:**
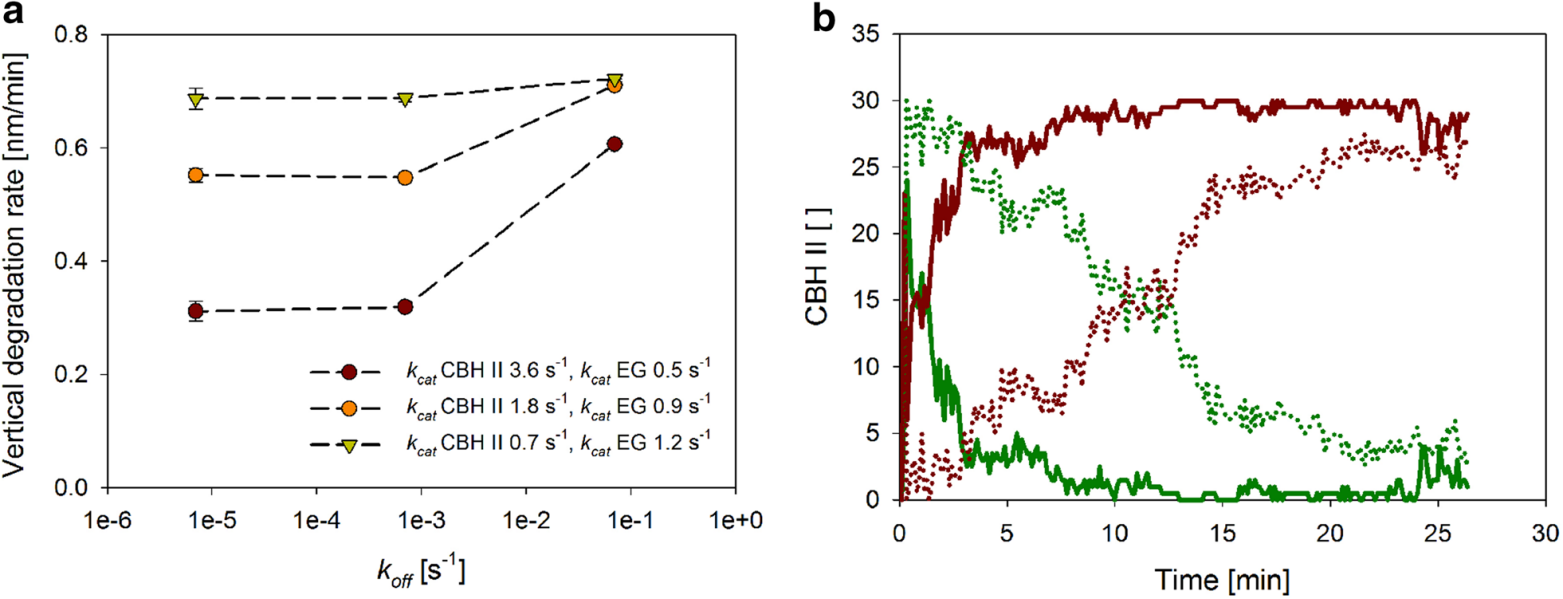
Degradation of amorphous cellulose by EG and CBH II. Different combinations of *k*_cat_ for EG and CBH II satisfy the criterion of the experimental *V*_z_ in amorphous cellulose, but only the combination *k*_cat_ (EG) > *k*_cat_ (CBH II) results in a plausible system behavior. **a** The value of *k*_off_ affects *V*_z_ strongly when *k*_cat_ (EG) < *k*_cat_ (CBH II), whereas it does not affect *V*_z_ when *k*_cat_ (EG) > *k*_cat_ (CBH II). **b** Apparent inactivation of CBH II occurs as result of switch of the enzyme into a resting state on collision with structural obstacles or other enzymes. *Green lines* indicate the fraction of active CBH II enzymes, while *red lines* indicate the fraction of resting CBH II enzymes. *Solid lines* met the criteria *k*_cat_ (EG) < *k*_cat_ (CBH II) and *dotted lines* met the criteria *k*_cat_ (EG) > *k*_cat_ (CBH II). The *k*_off_ for CBH II was fixed at 0.7 × 10^−3^·s^−1^. A relatively low *k*_off_ was chosen because the shown effect is particularly pronounced under these conditions (panel **a**)

Figure 5a shows that different combinations of *k*_cat_ for CBH II and EG were consistent with a *V*_z_ of about 0.7 nm/min, provided that the *k*_off_ for CBH II was very high. The different *k*_cat_ conditions tested in the simulations are summarized in Table 2. Interestingly, therefore, when the *k*_off_ was decreased, enzyme combinations having *k*_cat_ (CBH II) > *k*_cat_ (EG) showed a significant decline in the degradation rate whereby the effect was especially pronounced for the pair of relatively fastest CBH II and slowest EG (Fig. 5a). Lateral rather than vertical surface degradation became the predominant mode of substrate conversion in these cases, for the activity of CBH II progressively outweighed that of EG. The effect is understandable because under conditions in which the generation of new chain ends by the action of EG is limiting, the activity by CBH II becomes increasingly confined to the processive degradation of the cellulose chains already available on the surface. The overall rate of cellulose degradation also slows down as a result. The enzyme combination of relatively fast EG and slow CBH II exhibited a *V*_z_ approximately at target value that was however independent of *k*_off_ in the set range (Fig. 5a). Generally, under conditions in which the *k*_cat_ (EG) exceeds the *k*_cat_ (CBH II), the effect of the EG activity on reducing the free path length for the processive action of CBH II has the consequence that a more local and hence vertical degradation of surface material is favored. Suggestion from these simulations that *k*_cat_ (EG) should exceed *k*_cat_ (CBH II) was reinforced by evidence from statistical analysis of the functional state, processively chain-cleaving or inactively awaiting chain dissociation, the CBH II molecules were in during the simulated reaction. Figure 5b shows that for an assumed *k*_off_ for CBH II of only 0.7 × 10^−3^·s^−1^, the system featuring a high-*k*_cat_ CBH II rapidly went into a state where all of the CBH molecules simulated were just “resting.” Under conditions of a low-*k*_cat_ CBH II, by contrast, the CBH II molecules in the system remained in the actively chain-cleaving state for a substantially longer period of time Fig. 5b). Intuitively, therefore, one would favor the system with a comparably low *k*_cat_ for CBH II while refuting others that resulted in largely inactive CBH II populations. The *k*_cat_ of EG was thus estimated at about 1.2 s^−1^ and that of CBH II at about 0.7 s^−1^. Compared to literature [46, 64], the *k*_cat_ of EG is relatively small, being lowered by a factor between 2.9 and 6.8. The differences in *k*_cat_ probably arise from the different cellulosic substrates used. In addition, this study used room temperature (~20 °C) to perform the enzymatic reactions, whereas in literature 30 °C or higher was used.

**Table 2:** Summarization of the tested *k*_cat_ values for EG and CBH II and the resulting *V*_z_

*k*_cat_ [s^−1^]		*V*_z_ (nm/min) *without active CBH I molecules present*			*V*_z_ (nm/min) *with active CBH I molecules present*			
EG	CBH II	CBH II *k*_off_ [s^−1^]			CBH II *k*_off_ [s^−1^]			
		0.7 ×			0.7 ×			
		10^−1^	10^−3^	10^−5^	10^−1^	10^−3^	10^−5^	10^−2a^
0.9	1.8	0.71	0.55	0.55	–	–	–	–
0.9	1.2	0.64	0.55	0.54	–	–	–	–
0.6	3.6	0.65	0.37	0.37	–	–	–	–
0.5	3.6	0.61	0.32	0.31	–	–	–	–
1.2	1.2	0.77	0.70	0.71	0.83	0.73	0.75	–
1.2	0.9	0.75	0.70	0.69	0.81	0.74	0.73	–
*1.2*	*0.7*	0.72	0.69	0.69	0.77	0.73	0.73	*0.75* ^b^
1.2	0.6	0.71	0.69	0.68	0.75	0.73	0.73	–

Simulations were performed in the absence or presence of active CBH I molecules and the *k*_off_ of CBH II was varied at the values indicated. The values are shown as mean *V*_z_ (nm/min). The relative SD is below 5 % in all cases and therefore not shown. Simulations were performed in triplicates and over a time of 1575 s. Simulations matching the condition *k*_cat_ (EG) < *k*_cat_ (CBH II) were not tested with active CBH I molecules (see *Estimation of the k*_*cat*_ *of EG and CBH II*). The finally chosen *k*_cat_ pair and the results obtained with an optimized *k*_off_ of CBH II are highlighted

^a^Optimized *k*_off_ of CBH II

^b^Mean value of *V*_z_ is not affected by the implementation of *k*_endo_ for CBH II

We proceeded by introducing *k*_endo_ into the CA model of CBH II and analyzed the *V*_z_ of the enzyme degrading a purely amorphous cellulosic substrate. The cellulose surface was saturated with CBH II. Using a *k*_cat_ of 0.7 s^−1^, the value of *k*_endo_ was iteratively varied until a *V*_z_ of 0.13 ± 0.02 nm/min was obtained. The *k*_endo_ was thus estimated as 0.03 s^−1^. We show in Fig. 4c that the exo–endo model of CBH II gave a prediction of the time-dependent cellobiose release by the enzyme that agreed with the experimental data within a twofold range. The CBH II model was also able to describe conversion of the substrate in substantial amounts (>40 %), which the pure exo-model could not. The overall *V*_z_ applying all modeled cellulases was not significantly influenced by the assignment of *k*_endo_ to CBH II (Table 2).

A small piece of experimental evidence whose possible significance was not previously recognized [13, 14], namely that the presence of active CBH I weakly boosts the *V*_z_ of EG and CBH II, now turns out to be useful in the validation of the modeling results. Enhancement of *V*_z_ by about 3–10 % due to CBH I was reproduced by the model in good quantitative agreement (Table 2) with experiment. Despite its complete lack of activity on amorphous cellulose, the modeled CBH I affects the performance of EG and CBH II. It does so by virtue of removing crystalline substrate parts that restrict the unhindered access of the two other cellulases to the amorphous material.

### Degradation of crystalline fibrils within an amorphous cellulose matrix

**Fig. 6:**
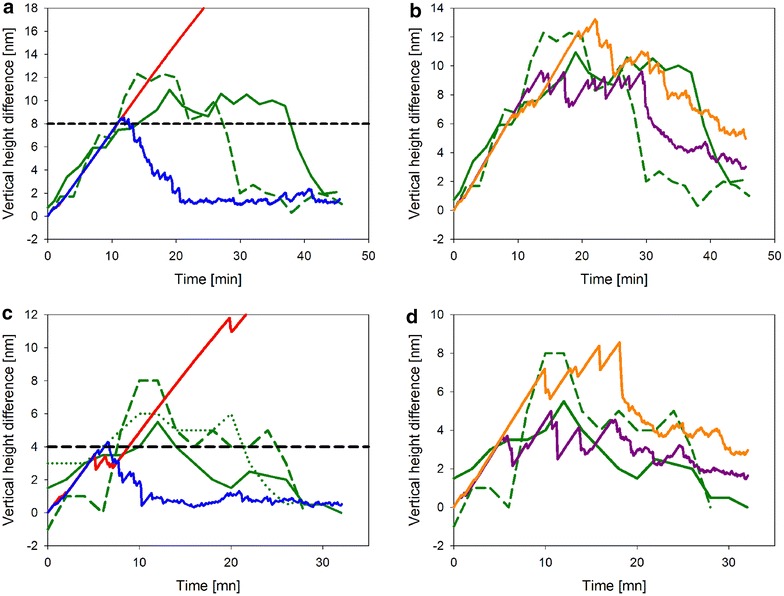
Dynamics of crystalline cellulose degradation depends on the *k*_off_ of CBH II. The *k*_off_ of CBH II is key to explain the dynamics of crystalline cellulose degradation by cellulases as modeled. Shown are time courses of ∆h_max (vertical height difference between the highest point on a cellulose crystallite and the amorphous material surrounding the crystallite) during enzymatic degradation of large (~16 nm height; panels **a**, **b**) and small (~8 nm height; panels **c**, **d**) nanocrystals analyzed by experiment and simulation. Nanocrystals were modeled with their hydrophobic faces aligned horizontally and with their top face touching the enzyme-accessible surface. The ∆h_max required to expose the crystal’s hydrophobic faces was therefore half the height, which is indicated by the *dashed black line*. Experimental time courses are shown in *green*. Simulated time courses of ∆h_max using low and high boundary values of *k*_off_ are shown in *red* (0.7 × 10^−3^·s^−1^) and *blue* (0.7 × 10^−1^·s^−1^), respectively. Shown in *orange* and *magenta* are two simulations using the iteratively “optimized” value of 0.7 × 10^−2^·s^−1^ for *k*_off_

Figure 6 shows time courses of ∆h_max determined from experimental AFM sequences in which concurrent degradation of cellulose nanofibrils and the amorphous cellulose matrix surrounding them was revealed. We note substantial variability in the results obtained from individual crystals, and trends of the ∆h_max values were certainly overlapped with considerable scatter. However, we think that some discontinuity in the ∆h_max curves is also intrinsic, as will be discussed later. Shared feature of degradation of relatively large (Fig. 6a, b; diameter 14–18 nm) and small (Fig. 6c, d; diameter 8–10 nm) nanocrystals was a triphasic time course in which ∆h_max increased gradually to a maximum value in the first phase, then stayed approximately constant for some time, only to drop off afterwards and vanish at the end of the process (∆h_max ~0). Degradation of large nanocrystals took longer overall and involved a higher ∆h_max change than degradation of the comparably small crystals (Fig. 6a, c). The rate of ∆h_max evolution in the first degradation phase was similar irrespective of the cellulose nanocrystal selected for analysis. Its average value of about 0.7 nm/min was consistent with the suggestion that it reflected primarily the removal of amorphous material surrounding the crystals. Figure 6a and c also shows that only after having been uncovered from the amorphous matrix at about half of their diameter, the nanocrystals started to become degraded. Interestingly, therefore, the clear evidence that ∆h_max decreased again in the final phase of the reaction (Fig. 6a, c) implies that the crystalline material was degraded faster than the amorphous cellulose.

Vertical degradation rates were estimated to differ by a factor of around five. The *V*_z_ of about 3.5 nm/min for degradation of crystalline cellulose was consistent with the results of Ganner et al. [14]. Applying the parameter values from Table 1, the CA model was capable of reproducing the dynamics of ∆h_max in useful qualitative and even (semi)-quantitative agreement with the experiment. First insight from the simulations however was that a time dependence of ∆h_max was observed only in the case that the nanocrystal was embedded in amorphous cellulose with its hydrophobic face buried. If in contrast the crystal’s hydrophobic face was exposed on the surface, as shown for the crystal in vertical orientation in Fig. 1a, degradation of the crystal was limited by removal of the surrounding amorphous cellulose, hence ∆h_max was zero throughout. The model prediction was consistent with AFM measurements [14], showing that at various places on the flat surface of the cellulose the material degradation did in fact involve removal of nanocrystalline phases without generating a significant height difference to the immediately surrounding amorphous matrix.

Modeling the degradation of nanocrystals in horizontal orientation where both hydrophobic faces were buried in amorphous cellulose revealed a pronounced dependence of the dynamics of ∆h_max on the *k*_off_ of CBH II, as shown in Fig. 6. Simulations performed at varied *k*_off_ for degradation of a large nanocrystal showed that change in *k*_off_ affected primarily the rate of ∆h_max decrease after the first phase of reaction (Fig. 6a, c). When the *k*_off_ was set too high (≥0.7 × 10^−1^·s^−1^), due to fast degradation of the crystal the emergence of ∆h_max was much smaller than observed experimentally. When by contrast the *k*_off_ was set too low (≤0.7 × 10^−3^·s^−1^), the nanocrystal was hardly degraded in the relevant time span of the simulation experiment. Of note, therefore, this finding implies CBH II to have a substantially higher *k*_off_ than CBH I. At the single-molecule level, the immediate consequence of lowering the *k*_off_ of CBH II was an increased tendency of enzyme jamming at the interface of crystalline and amorphous cellulose.

Because the analysis is performed at the single-nanocrystal level, and it has to be done in that way to capture local characteristics of the substrate degradation, the stochastic effects introduce large variability in the time courses of ∆h_max, both when degradation of the *same* nanocrystal is examined in multiple simulations or when degradation of *different* but similarly sized nanocrystals is studied experimentally. Figure 6 shows that both experiment and simulation yielded a relatively broad ensemble of ∆h_max time courses, and it was clear that averaging would not have eliminated the stochastic effects. However, despite this it was still possible to perform an iterative search for a *k*_off_ of CBH II that allowed the model to reproduce the overall trend of the data. Using the time courses for degradation of two large nanocrystals, as shown in panel b of Fig. 6, the *k*_off_ of CBH II was estimated to be about 0.70 (±0.10) × 10^−2^·s^−1^. Thus, simulated time courses of ∆h_max were well consistent with the experimental results, except for the systematic difference that the ∆h_max at the end of the nanocrystal degradation cycle did not drop to zero (Fig. 6b). In the simulation, due to the constant *V*_z_ in amorphous material surrounding the nanocrystal, a height difference develops inevitably between this material and the amorphous material underneath the crystal. The height difference is eliminated only gradually in the simulation as result of a slightly elevated *V*_z_ in the protruding material, which offers a larger enzyme-accessible area than the flat surfaces adjacent to it. Effects involved in the experiment but not accounted for in the CA model are not clear at this time. However, not necessarily it is implied that the CA model of cellulase action presents an oversimplification. It is also possible that the small surface structures in amorphous material arising in the simulation after the nanocrystal has become degraded are not stable in the experiment, e.g., because they are only loosely attached to the surrounding material which might even result in their removal by the AFM tip.

In a final step, the dataset for small nanocrystal degradation was used for model validation. Results are shown in Fig. 6d. The high and low boundary values for *k*_off_ were completely inconsistent with the experimental evidence, as indicated in panel C of Fig. 6. Using the “optimized” *k*_off_, however, useful trend agreement between simulated and experimental time courses was obtained (Fig. 6d). Moreover, for both small and large nanocrystals, the *V*_z_ of crystalline material degradation of about 3.1 ± 0.1 nm/min was predicted in almost perfect accordance with the experimental observations.

### CA modeling reveals single-molecule dynamics of degradation of amorphous–crystalline cellulose

**Fig. 7:**
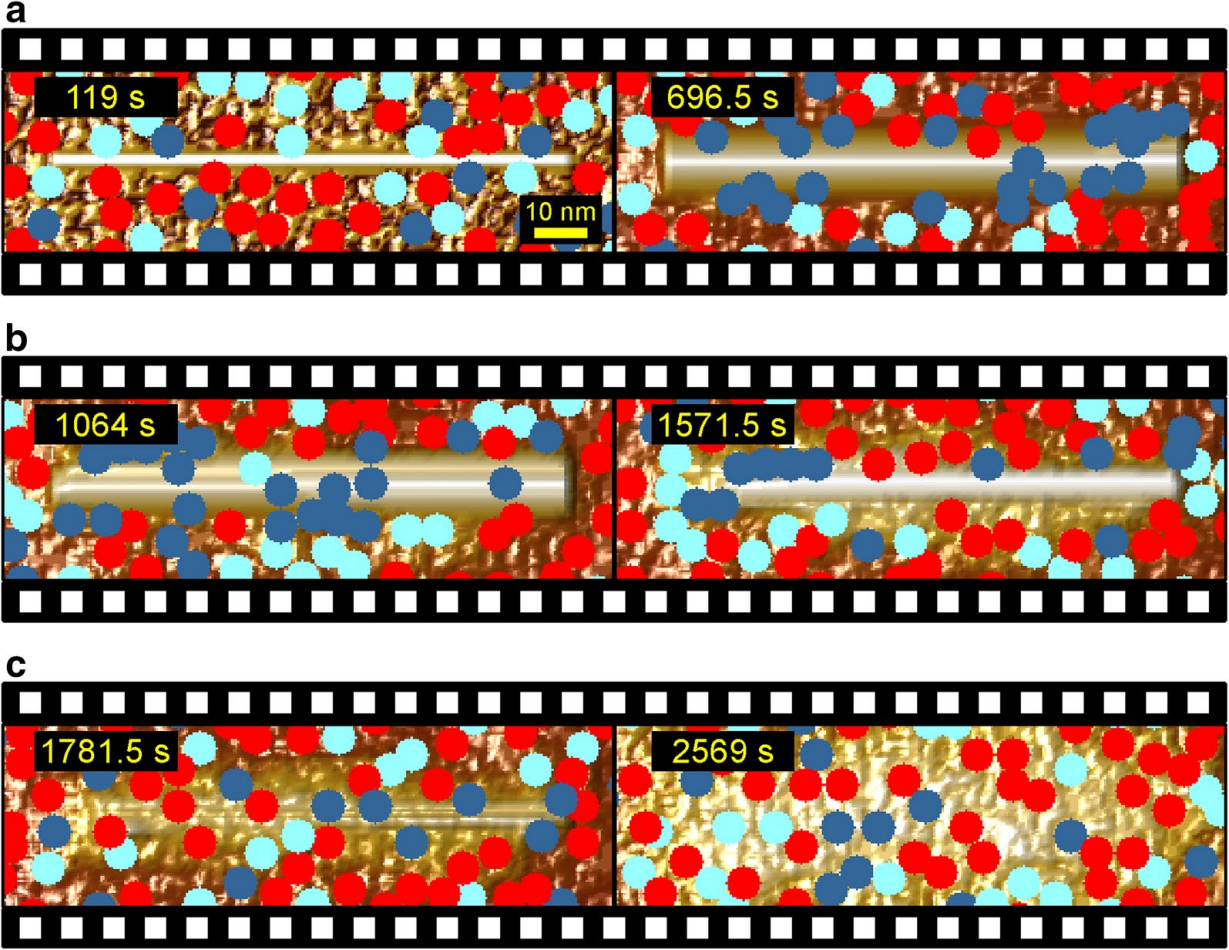
Time-resolved sequences from a simulated degradation of mixed amorphous–crystalline cellulose by the cellulase system. Cellulases are identified by *color* EG (*red*), CBH II (*light blue*), and CBH I (*dark blue*). **a** A crystallite is gradually uncovered over time until the horizontally oriented hydrophobic face is revealed. **b** Upon revealing of the hydrophobic face, the crystallite is attacked in an asymmetric manner. **c** Finally, the crystallite becomes completely degraded over time. A video of the shown degradation sequence is available (Additional file 2). Please note that occasional visual overlapping of enzymes is caused by their position in different *z*-planes of the substrate

**Fig. 8:**
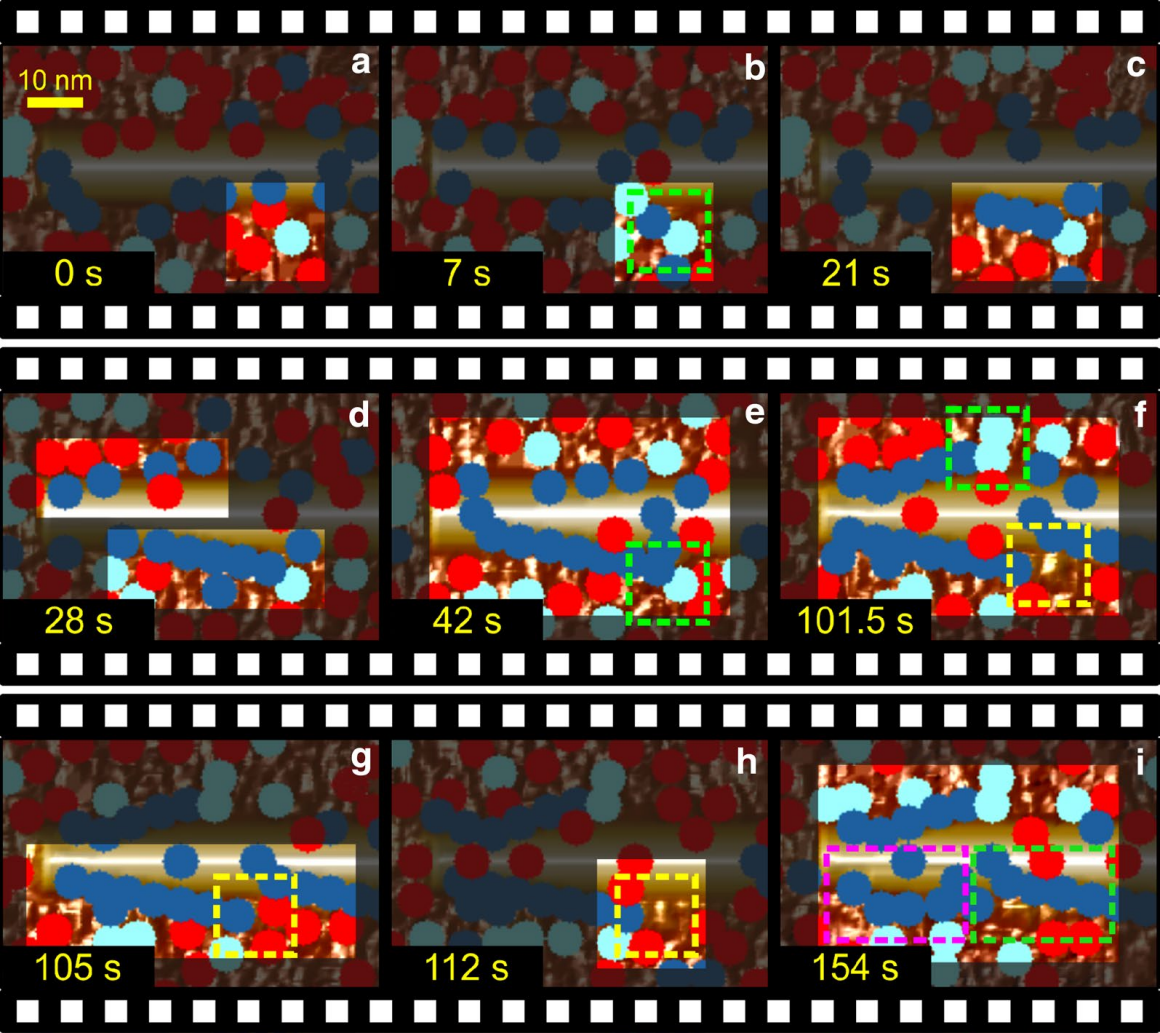
Dynamic formation and dissipation of enzyme traffic jams at the amorphous–crystalline interface. Cellulases are identified by *color* EG (*red*), CBH II (*light blue*), and CBH I (*dark blue*). For an easier viewing, the background is darkened through all panels. **a**, **b** An already-trapped CBH II molecule at the interface of crystalline and amorphous cellulose (*green rectangle*) causes a traffic jam of CBH I molecules. **c** CBH I molecules move processively from left to right on the upper edge of the crystal and an accumulation of CBH I can be observed (21 s). **d**, **e** Degradation starts on the other site of the crystallite too but is soon stopped by another collision with a CBH II molecule (*green rectangle*, panel **f**). **f** After 1/*k*_off_ is passed, CBH II dissociates but the amorphous material below the dissociated CBH II is clearly elevated in comparison to the surrounding amorphous material (*yellow rectangle*). Note: the elevated height is recognized by *bright color* in the *yellow framed area*. The lower lying surrounding material is indicated by (*dark*) *brown color*. **g**, **h** EG molecules attack the (elevated) amorphous part (*yellow rectangle*) and clearly alter it by reducing its height. **i** Eventually, CBH I molecules resume hydrolysis (*green square*, 154 s). However, the next group CBH I enzymes trying to slide along the crystal on a lower plane are trapped again (*pink square*). Note that, the crystallite shows already degradation (*pink square*) caused by the first group of enzyme sliding along. A video is available (Additional file 3)

A full video of a simulated degradation of a large nanocrystal embedded in amorphous material is provided (Additional file 2). The video illustrates the main dynamic features of enzymatic cellulose surface degradation revealed in this study. At the mesoscopic level, time-dependent evolution and decay of a height difference between crystalline and amorphous material is shown (Fig. 7a–c). At the level of the single enzymes, the repeated occurrence of cycles of formation and dissipation of enzyme jams on crystalline cellulose is demonstrated. Snapshots from the video are shown in Fig. 7 to demonstrate the effect, revealing that stochastic processes determine when and where the jams occur. Jams of CBH I molecules moving along the surface of crystalline cellulose in repeated stop-and-go cycles have been observed in experiments before [16]. These jams were however intrinsic to the action of CBH I. What is unexpected and new here, as emphasized in Fig. 7 and in more detail in Fig. 8, is the suggestion that jams of CBH I caused by trapped CBH II molecules might play a decisive role in determining the dynamics of degradation of amorphous–crystalline cellulose material.

The dynamics of formation and dissipation of enzyme jams caused by stuck CBH II molecules is clearly recognized in Fig. 8 and Additional files 2 and 3. CBH II molecules awaiting chain dissociation represent obstacles for processively acting CBH I molecules, and this results in an intermittent stopping of cellulose degradation in the crystalline surface areas affected by the jam (Fig. 8a–c) and an accumulation of trapped CBH I molecules (Fig. 8b–e). Because surrounding amorphous material is degraded rather continuously, degradation of the crystal in discrete stop-and-go phases results in a discontinuous up-and-down trend of ∆h_max within the time domain of 1/*k*_off_. This was observed experimentally and also reproduced by the model (see Fig. 6). Jamming events caused by CBH II are intrinsically stochastic in consequence of both the random structure of the amorphous material and the random diffusion of uncomplexed CBH molecules on the surface.

Also due to stochastic effects in the simulation, it was possible that traffic jams occurred predominantly on one side of the cellulose crystal or in temporally delayed fashion (compare Fig. 8b, f), thus resulting in a pronouncedly asymmetric degradation of the crystal from the side opposite to the jam. This result was interesting because similar patterns of asymmetric degradation of cellulose crystals were noted in AFM imaging experiments [14].

### CA modeling suggests “hidden” morphological targets for cellulase synergy in the degradation of crystalline cellulose

It was earlier suggested from experiment [22, 46] that efficiency of CBH I for degradation of different celluloses is limited by the mean free path length of the enzyme’s processive chain cleavage. Simulations made herein revealed that obstacles from surrounding amorphous cellulose restricted the processivity of CBH I on crystalline substrate. Removal of the obstructing material by the partner cellulases, in particular EG, restored the processive action of CBH I and thus enhanced the free path length of chain cleavage for the enzyme. Figure 8e, f shows an example where a CBH II molecule that had become temporarily stuck right next to the cellulose crystal prevented degradation of amorphous material lying underneath or in immediate proximity to it. Therefore, this resulted in a local increase in amorphous height in the surface region affected. After the release of the trapped CBH II, the protruding amorphous surface constituted a structural obstacle for attack of CBH I on the cellulose crystallite. As a consequence, additional CBH I traffic jams were created and complete crystalline chain degradation toward the non-reducing end was prevented (Fig. 8f–h). Only after removal of the obstacle, here through attack by EG molecules (Fig. 8g, h) did the degradation of the crystal resume, as shown in Fig. 8i. It is worthy of note that similar structural obstacles could arise anytime during the simulated reaction also in the absence of a stuck CBH II molecule, for they represent an intrinsic feature of the spatiotemporal dynamics of surface degradation by the CA model based on random principles. These obstacles are identified as clear morphological targets for synergy between amorphous material-degrading cellulases and CBH I. Moreover, a new potential role of CBH II and EG in facilitating degradation of crystalline cellulose by CBH I is suggested. Besides uncovering cellulose crystals from amorphous material to make attack by CBH I possible in the first place, the two cellulases are also required to remove dynamically appearing local obstacles of amorphous cellulose that obstruct the processive action of CBH I. Overall, therefore, CBH II and EG contribute to enhancement of the mean free path length of processive chain cleavage by CBH I, and this is expected to be beneficial for efficient substrate degradation as a whole.

### CA modeling the release of soluble sugars

**Fig. 9:**
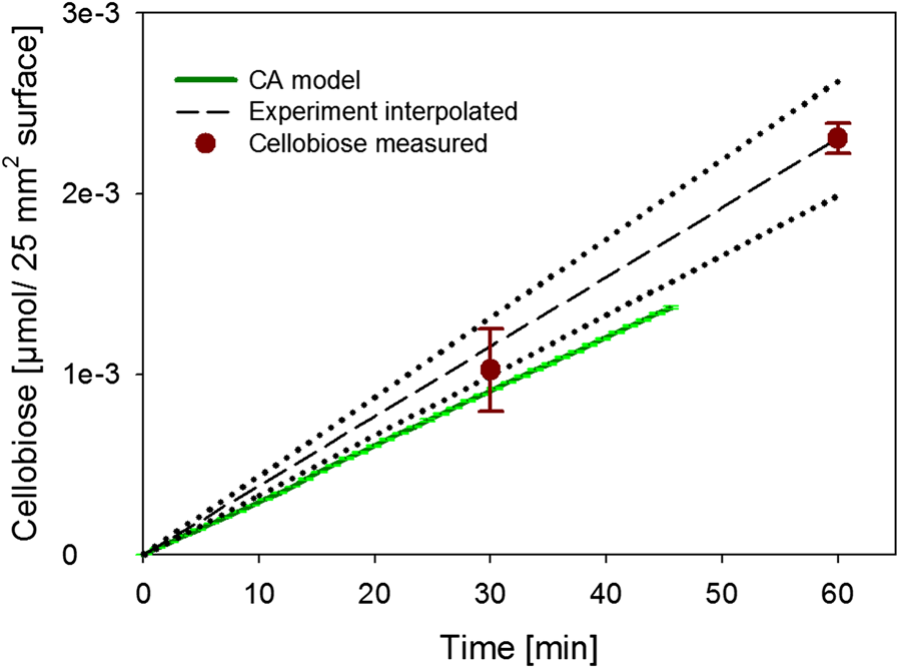
Modeled time course of cellobiose formation is compared to experiment performed under exactly comparable conditions. The experimentally measured initial production of glucose is expressed as cellobiose released (glucose release/2). The absolute amount of calculated cellobiose is plotted as a function of released cellobiose from 25 mm^2^ cellulose surface with *black dashed line* and the range of error is shown as *dotted line*. Experimentally measured points are indicated in *red*. All experiments and simulations were conducted at least in triplicates

Knowing that prominent features of cellulose surface degradation were represented realistically in the simulations, it was interesting to examine the CA model’s capability of predicting the soluble product release. Figure 9 compares time courses of cellobiose formation in experiment and simulation, showing that the model only slightly (by about 30 %) underestimated the experimental cellobiose release. Given the model’s complexity plus the fact that parameters of enzyme action were both realistic and not excessively fine-tuned to match the experiment, the CA modeling can be considered to have provided a very close prediction of the actual hydrolysis rate. From the sugar release rate and the amount of total protein adsorbed, a specific activity of the bound “cellulase” of 1.77 ± 0.05 µmol/(min mg_protein) was calculated.

### Advances made in CA modeling of the enzymatic cellulose degradation

The current CA model of enzymatic cellulose degradation is unique in having been designed for effective use in combination with time and laterally resolved experimental data from high-resolution imaging analysis. An important novel feature was the CA model of a mixed amorphous–crystalline substrate whereby the amorphous material was built from surface layers of completely randomly organized cellulose chains. Previous CA models have focused mostly on crystalline cellulose and represented its ordered structure at different levels of detail [31, 36, 43]. Coarse-grained models of crystalline cellulose were applied to simulate pretreatment effects on the substrate structure [65, 66] or to construct potential free energy surfaces for an atomistically represented cellulose-binding module [67]. Kumar and Murthy [37] included regions of amorphous material in their 3D matrix model of a microfibrillar cellulose structure. However, in their model the amorphous regions were created from single cellulose chains that alternated between being embedded in the crystalline fibril structure or being released from it. Disorder in direction and orientation of the cellulose chains was therefore not modeled. However, the evidence from the current modeling work supports the suggestion that the interface between crystalline and (randomly organized) amorphous material constitutes an important region of high local dynamics in substrate morphology and in the correlated synergistic interplay of the cellulases.

With only one (EG) or two (CBH I, CBH II) main adjustable parameters (*k*_cat_, *k*_off_), the herein used CA model of enzyme action confined to the cellulose surface was simple and focused. CA models in the literature usually included enzyme adsorption and desorption additionally [31, 36, 43]. Surface diffusion was implemented in some [30, 36, 43] but not all models [31, 37]. A few studies tried to correlate the results of their modeling with experimental data of soluble sugar release [36, 37]. However, the experimental substrate was usually very different from the substrate modeled (e.g., Avicel compared to a cellulose fibril [37]), thus making a direct comparison challenging.

## Conclusions

CA modeling is shown to depict realistically the degradation of mixed amorphous–crystalline cellulose by a three-enzyme-type cellulase system. Total system behavior is successfully reconstructed by combining kinetic data sets from high-resolution single-molecule studies. Most noticeable, salient dynamic features of cellulose surface degradation by different cellulases acting in synergy are reproduced and close prediction of the actual hydrolysis rate is thus achieved. Results of the modeling can be analyzed visually and are therefore ideal for comparison with the evidence from high-resolution visualization experiments. Moreover, the modeling approach employed in this work illustrates the importance of spatial resolution at single-molecule level in both in situ and in silico studies. Besides aiding in the mechanistic interpretation of intrinsically complex effects from the experiment, the modeling also provides deepened insight into the possible interplays, some of them of a stochastic nature, of individual cellulases during cellulose degradation. The model thus helps making explicit what is usually only implicit in experimental observations of enzymatic cellulose hydrolysis. In particular, the *k*_off_ of CBH II is suggested here for the first time to have a key role in maintaining the dynamics of action of the processively cleaving CBHs at the interface of amorphous and crystalline cellulosic material. The current model is expandable. More complex substrate structures can be implemented. New enzyme properties can be defined, to incorporate non-productive binding to the cellulose or a finite enzyme lifetime due to inactivation for example. Enzyme adsorption dynamics at subsaturating enzyme loading can also be included. It would also be of interest to implement surface diffusion over distances greater than simulated here. Using simulation, the impact of change at the single-molecule level on total system behavior can thus be studied. Insights obtained from CA modeling are of fundamental significance but also relevant for practical application in enzymatic cellulose hydrolysis.

## Methods

### Programming, simulation, and data processing

All programming was done in MATLAB [Version 7.11.1.866 (R2010b) service pack 1]. The complete program code is available on request. A Monte Carlo approach was used and individual steps are discussed in detail in “Results and discussion” section. Simulations were performed in a cuboid box with dimensions of 70 × 150 × 100 nm for the *x*, *y*, and *z* (height) coordinates, respectively. The cellulose substrate was modeled as an initially completely flat surface that was continuous (i.e., did not contain walls) in the *x* and *y* direction. Cellulosic planes were assembled on top of each other in the *z* direction, making up a material layer of up to 90 nm.

Crystalline phases were included as cellulose nanofibrils. For clarity reason, the nanofibrils were oriented always in plane parallel to the cellulose surface. Their hydrophobic faces were aligned horizontally or vertically, as shown in Fig. 2a. Any orientation of the nanofibril is however possible in the model. A water phase (Fig. 1) was included to indicate the accessible cellulose surface.

To mimic the experimental conditions of AFM imaging in which completely saturating cellulase loadings (4 × 10^4^ mg protein/m^2^ cellulosic surface) were applied in solution, all simulations were performed at an assumed maximum surface coverage with enzyme of 24 nmol/m^2^. Lying in between the maximum cellulase adsorption capacity estimated from a “two-dimensional liquid crystal” approximation of the adsorbed cellulase (16 nmol/m^2^; [68]) and the one determined experimentally by Maurer et al. [68, 69], the assumed surface loading appeared to be realistic. The composition of the adsorbed cellulase was set approximately according to the substrate sites available in the cellulose as 60 % EG and 20 % of each CBH. The total number and the composition of the cellulases were invariant during the simulation. The enzyme kinetic parameters from Table 1 were used. Considering the involvement of multiple random steps in the CA model of cellulase action, simulations were performed in at least 10-fold replicates to obtain statistic validation. Parameters obtained from simulation data are therefore provided as mean values with standard deviation.

Reaction time courses were recorded as movies of cellulose surface degradation. These movies were used for qualitative comparison with time-resolved evidence from AFM imaging. The rate of height removal in amorphous material and also the rates of emergence and degradation of crystalline nanofibrils could be compared quantitatively between experiment and simulation. Cellobiose released from the surface was also calculated and compared with experiment. Parameter used to express enzymatic action of the crystalline material was the time-dependent height difference between the highest point on the crystal and the mean height of surrounding amorphous material (∆h_max). Because the rate of degradation of crystalline was highly dependent on the nanofibril’s aspect ratio, we modeled crystals of large (14–18 nm) and small (8–10 nm) diameters.

### AFM imaging and data analysis

**Fig. 10:**
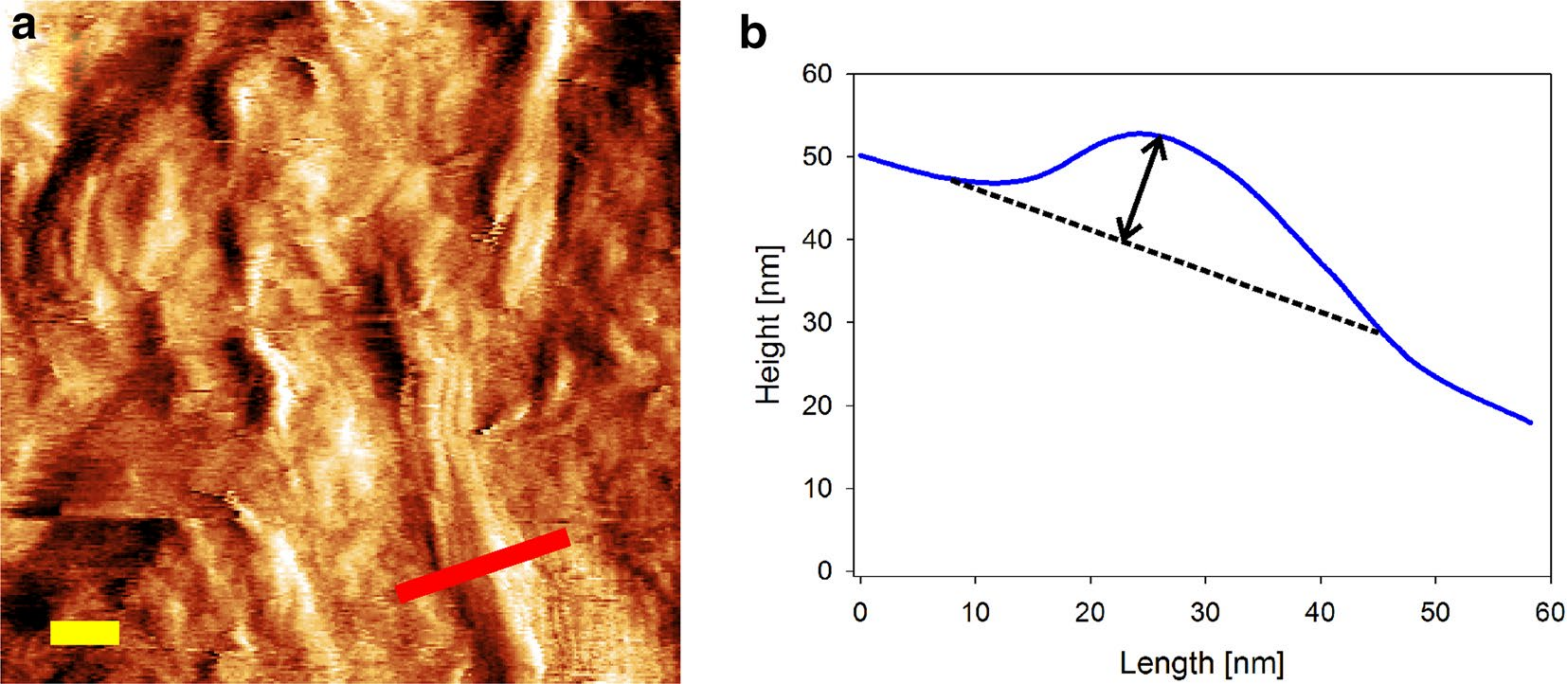
Exemplary processing of AFM images of enzymatic degradation to determine Δh_max. Crystalline fibrils embedded in amorphous cellulose matrix provide time-resolved quantitative data for comparison of simulation results with the experiment. **a** Height profiles (*red line*) of crystals were taken from AFM sequences recorded from enzymatic reactions carried out as described by Ganner et al. [14]. For easier viewing, the amplitude image of the corresponding position is shown. The *yellow scale bar* shows 10 nm. **b** The highest point on a cellulose crystallite was found by moving the section profile along the crystallite, and the vertical height difference between the highest point and the amorphous material (indicated by the *arrow*) surrounding the crystallite was calculated (∆h_max)

Experimental data were from our recent studies where in situ AFM imaging was used to monitor surface degradation in a nanoflat mixed amorphous–crystalline substrate [41]. In that way, the time-dependent change in ∆h_max could be determined. More specifically, section profiles were taken from an individual nanofibril at each time to determine the highest point in relation to surrounding amorphous level (Fig. 10). The values of ∆h_max are color-coded to match the color code used for the height measured in the AFM imaging experiments. Additionally, the AFM data were used to calculate at different points of the surface the vertical degradation of amorphous cellulosic material. A large micron-sized crystallite that was completely resistant to enzymatic degradation in the time span of the experiment was used as a height marker [14, 70]. AFM image processing and analysis was performed using Gwyddion 2.31 (released 02/21/2013) and Nanoscope (Build R3Sr4.94136, Bruker Nano Surface Offices). However, to improve the manual processing of multiple images, an automated routine in MATLAB was developed with which the images from an AFM sequence could be matched one on another in an exactly comparable fashion.

### Enzymatic hydrolysis

Experiments were performed in triplicates at 20 °C in sodium acetate buffer (pH 5.0). The mixed amorphous–crystalline cellulose applied to AFM studies was used. The substrate concentration was 2.0 mg/mL and the total reaction volume was 500 µL. Substrate was cut into pieces with dimensions of approximately 5 × 5 × 0.2 mm. Based on this geometry, an initially available surface area of 54 mm^2^ was calculated.

Reactions using the complete *H. jecorina* cellulase system contained 25 µg protein/mg cellulose. β-Glucosidase (Megazyme, Wicklow, Ireland) was added at 5 µg protein/mg cellulose. Reactions using purified CBH II alone also contained 25 µg protein/mg cellulose and β-glucosidase (2 µg/mg protein). Samples were taken at suitable times and analyzed for d-glucose using glucose oxidase and peroxidase [70]. d-Glucose concentrations below detection limit of the enzymatic assay were analyzed by high-performance anion-exchange chromatography with pulsed amperometric detection [13]. To compare experimental hydrolysis data with the modeling results of soluble sugar release, the amount of cellobiose formed in the experiment was assumed to be half that of the d-glucose measured. The µmol amounts of cellobiose released per 25 mm^2^ surface were compared.

## Additional files

10.1186/s13068-016-0463-8 Exemplary construction of the chain orientation matrix of one amorphous cellulose plane. Cellobiose units not yet oriented in plane are shown in green. Otherwise the colour coding of Fig. 2b is used. The orientation (*x*, *y*) and internal direction (‘+’, ‘−’) of each chain with respect to the origin of the *z*-plane are indicated by colour: orange (*x*; +1), light blue (−*x*; −1), red (*y*, +2) and dark blue (−*y*, −2). A time-efficient multistep algorithm was designed to saturate an amorphous cellulose plane with fragments mimicking a Gaussian distribution. Chains are positioned by the following procedure: at first, cellulose chains with random length according to a normal distribution (*μ* 15 nm, *σ* 3 nm) are generated and randomly positioned and oriented in the layer. Note: one cellobiose automaton is 1 nm. The possibility for chain positioning at first try decreases significantly with progressing surface coverage until a limiting surface coverage of about 80 % is reached (Additional file 1, 0–19 s). The remaining 20 % of the surface are filled as follows: existing cellulose chains are elongated in a random process by attaching terminal cellobiose units without orientation (green) to the chain (Additional file 1, 20–30 s). Thus, the elongation process can be applied until a surface coverage of above 95 % is reached. In the case that cellobiose units without an orientation are encapsulated and no chain end terminates in direction of the not yet oriented cellobioses (green), shorter chain fragments with a random orientation are used to fill the area (Additional file 1, 31–34 s). Note, that changing the orientation of already oriented cellobiose units is widely prohibited during all steps. However, to achieve a 100 % surface coverage in a reasonable time (<30 s per plane) exceptions to this rule are implemented in the algorithm. These exceptions cause a negligible deviation from the normal distribution (see Fig. 2c). A mean free path length of 18 nm is reached eventually.
10.1186/s13068-016-0463-8 Time-resolved sequences from simulations of mixed amorphous-crystalline cellulose degradation by the cellulase system. Cellulases are identified by color: EG (red), CBH II (light blue), CBH I (dark blue). A crystallite is gradually uncovered over time until the horizontally orientated hydrophobic faces are exposed (Additional file 2, 0–60 s). Upon revealing of the hydrophobic faces CBH I molecules are moving processive along the crystallite (Additional file 2, 60–65 s) thereby exposing new hydrophobic sites (see Fig. 2a). A typical sequence of events hindering CBH I molecules in their possessive motion is initiated after 68 s in Additional file 2. This sequence includes CBH I molecules jamming with a CBH II molecule (Additional file 3, 68–76 s) and later with amorphous material (Additional file 2, 78–84 s). This sequence is shown in more detail in Fig. 8 and Additional file 3. Similar events of CBH I molecules blocked by CBH II molecules or amorphous obstacles can be observed until the crystallite is completely degraded (Additional file 2, 170 s). The video is speeded up by a factor of 10.5.
10.1186/s13068-016-0463-8 Details of dynamic formation and dissipation of enzyme traffic jams in a simulated degradation of mixed amorphous-crystalline cellulose by the cellulase system. Cellulases are identified by color: EG (red), CBH II (light blue), CBH I (dark blue). Initially, an already trapped CBH II molecule at the interface of crystalline and amorphous cellulose causes a traffic jam of CBH I molecules and an accumulation of CBH I molecules (Additional file 3, 0–10 s). After 1/*k*_off_ is passed, CBH II dissociates but the amorphous material below the dissociated CBH II is clearly elevated in comparison to the surrounding amorphous material (see Additional file 3, 14 s). Thus, not all CBH I enzymes are allowed to proceed with their processive motion (Additional file 3, 10–15 s). Multiple EG molecules attack the (elevated) amorphous part and alter it by reducing its height and thinning it (Additional file 3, 15–21 s). Eventually, CBH I molecules resume hydrolysis (Additional file 3, 21–23 s). However, the next group CBH I enzymes trying to slide along the crystal on a lower plane are trapped again (Additional file 3, 24–27 s). The highlights of this process are depicted in Fig. 8. The video is speeded up by a factor of 7.

## Abbreviations

CA: cellular automata
AFM: atomic force microscopy
CBH: cellobiohydrolase
EG: endoglucanase
*V*_z_: vertical degradation rate
